# Environmental stochasticity and intraspecific competition influence the population dynamics of *Culex quinquefasciatus* (Diptera: Culicidae)

**DOI:** 10.1186/s13071-018-2711-1

**Published:** 2018-02-27

**Authors:** William T. Koval, Gonzalo M. Vazquez-Prokopec

**Affiliations:** Department of Environmental Sciences, Emory University, Atlanta, GA 30322 USA

**Keywords:** Density dependence, Population modeling, *Culex quinquefasciatus*, Vector management

## Abstract

**Background:**

Members of the *Culex pipiens* complex (*Cx. pipiens quinquefasciatus* in Southern USA) play a critical role in the spillover of urban arboviruses such as West Nile virus or St. Louis encephalitis virus. Field studies have shown strong correlation between the periodicity of rainfall events and larval proliferation. However, mechanistic determinants driving this relationship are poorly understood. We hypothesize that rainfall events decrease strain from intraspecific competition through the associated reduction of immature density and the introduction of detritus.

**Results:**

To address our hypothesis, we used laboratory competition experiments to inform a deterministic matrix projection model consisting of an age-structured larval matrix coupled with a stage-structured adult mosquito matrix*.* Rain events were simulated in a competition-based metabolic age model and compared to a null model including environmental variability. Variable rain delays in two-event simulations showed optimal proliferation occurring with rain delays between 16 and 21 days when including density-dependent effects.

**Conclusions:**

These results are comparable to the pattern observed in natural populations, indicating that *Cx. quinquefasciatus* proliferation rates can be modeled mechanistically as a density-dependent system. The empirical understanding of density-dependence as it relates to environmental stochasticity provides a theoretical platform for the study of larval dynamics and the impact of larval control in this medically relevant disease vector.

**Electronic supplementary material:**

The online version of this article (10.1186/s13071-018-2711-1) contains supplementary material, which is available to authorized users.

## Background

In the USA, members of the *Culex pipiens* complex (predominantly *Cx. pipiens* and *Cx. quinquefasciatus*) have been identified as the principal vectors of flaviviruses such as West Nile virus (WNv) and St. Louis encephalitis virus (SLEv) [1–3]. *Culex quinquefasciatus* is considered the principal human WNv spillover vector in southern USA due to its high abundance, competence, and infection rates [1, 3–7]. Although *Cx. quinquefasciatus* populations have been the target of intense vector control, particularly once symptomatic human WNv cases are detected, realized treatment efficacy remains difficult to demonstrate [8–10]. In addition to application cost, vector management in the *Cx. quinquefasciatus* system is limited by immature larval habitat characteristics [9, 11–13], fine scale heterogeneity in virus transmission [7, 14], and high levels of uncertainty in the prediction of disease outbreaks.

*Culex quinquefasciatus* requires a higher quality and variety of dissolved organics for survivorship to adulthood than do other mosquito species [15–18]. As *Cx. quinquefasciatus* oviposit egg raft masses of approximately 200 coeval individuals [19], selection of high nutrient environments is a key factor for successful larval mosquito development [17, 20, 21]. *Culex* spp. mosquitoes are commonly found in high numbers in artificial containers, unattended pools, retention ponds, storm drains, catch basins, sewage systems and pit latrines [12, 13, 22–28]. The high nutrient loading of such habitats limit predator survival and release *Cx. quinquefasciatus* from interspecific competition, leading to high larval densities that can become potentially vulnerable to intraspecific competition.

Culicine individuals subjected to higher larval densities exhibit delayed adult emergence, decreased fecundity, poor and uneven growth, and overall lower survivorship [12, 22, 29–32]. It is assumed that *Cx. quinquefasciatus* mass accumulation follows other insect models in that consumption rates rise with biomass while efficiency of mass conversion decreases due to higher metabolic rates [33, 34]. From this general relationship, it can be predicted that overall nutrient consumption of immature *Cx. quinquefasciatus* depends upon the population age structure and that, in order to maintain the commonly observed high densities of larval habitats, nutrient loading must be substantial and frequent.

Rainfall events provide water and nutrient resources to larval habitats, particularly urban catch basins, leading to immediate and delayed effects on larval mosquito proliferation [35]. Despite the flushing of culicine eggs and pupae from flooded habitats [36], larval proliferation within habitats has been shown to increase on average four days after a rain event [12, 37–39]. Adult proliferation has also been demonstrated to be time-lag associated with rainfall events. In Chicago, IL, approximately two weeks are required for oviposition and hatching to establish adult emergence peaks and an optimal periodicity of three weeks between rainfall events has been shown to increase the magnitude of these peaks [37, 38]. Despite empirical knowledge that rainfall events impact *Cx. quinquefasciatus* proliferation, the biological mechanisms driving this relationship are poorly understood and rarely considered within mathematical models [35, 37–40].

In this study, we linked larval competition experiments with mathematical models to understand the mechanistic basis of the association between rainfall and *Cx. quinquefasciatus* population dynamics. Our research hypothesis is that *Cx. quinquefasciatus* intraspecific larval competition is the driving mechanism of demographic responses, both immediate and latent, to rainfall regimes. To address this hypothesis, we first experimentally quantified the contribution of nutrient loading and population density to the larval dynamics of *Cx. quinquefasciatus*. Experimental data then informed a deterministic mathematical model exploring the interactive effects of rainfall, nutrient loading, and density-dependence on *Cx. quinquefasciatus* population size.

## Methods

### Life table parameter estimation

Field measurements and controlled experiments allowed quantification of natural fecundity and the influence of nutrient availability on immature life history traits, respectively. Fecundity was estimated by collecting naturally occurring eggs from Baker Woods (an urban forest patch located in Emory University, Atlanta, GA [17]) following the methods described by Chaves et al. [23]. Briefly, oviposition traps were left overnight and culicine egg raft masses were collected the following morning. A total of 40 coeval egg rafts were used for fecundity and fertility estimation. The number of eggs per raft was counted using a stereomicroscope (Leica IC80, Leica Microsystems, Wetzlar, Germany). After counting, rafts were placed individually inside a polystyrene vial with water to allow for larval hatching inside an insect growth chamber (6025–1, CARON Products, Marietta, OH, USA) set at 20 °C and 80% relative humidity, RH. Fertility was calculated for each egg raft by dividing the total larvae hatched by the total number of eggs. Four larvae from each raft were kept until fourth-instar to later identify egg rafts to species [41].

The effect of nutrient availability on immature life history traits (survivorship, time to stage change and consumption rate) was measured by subjecting *Cx. quinquefasciatus* larval cohorts to a four-level nutrient gradient (Table 1). Nutrient levels were informed by a pilot study that found no density-dependent effects within the high nutrient treatment and significant (> 95%) mortality within the low nutrient treatment.

**Table 1:** Nutrient concentration levels used for all experiments estimating life-table parameters

Treatment ID	Starting nutrient concentration	Starting nutrient availability	Stock added daily
High	0.3 mg/ml	120 mg	9 mg
Mid-High	0.15 mg/ml	60 mg	4.5 mg
Mid-Low	0.075 mg/ml	30 mg	2.25 mg
Low	0.0375 mg/ml	15 mg	1.125 mg

This design was replicated five times over the four treatments. Twenty mosquito breeding chambers (1425DG, Bioquip, Rancho Dominguez, CA, USA) were filled with 400 ml of untreated water from Peavine Creek (an unpolluted stream running close to Emory Campus). A stock nutrient solution was created from 300 mg of baker’s yeast (B2C, LeSaffre, Marcq-en-Baroeul, France) and 300 mg fish food (971,168, Carolina Biological Suuply Co, Whitsett, NC, USA) dissolved in 0.9 L of creek water (1.5 mg nutrients/ml) and diluted to match nutrient treatment levels at consistent volume (Table 1).

Forty first-instar larvae, collected from four different egg rafts (10 larvae per raft), were placed in each breeding chamber approximately 24 h after hatching. Breeding chambers were stored inside the insect growth chamber (20 °C, 80% RH) and mosquito development was monitored daily. Development was measured by counting and removing shed IV instar molt casings (time to pupation) and pupal molt casings (time to emergence). Daily mortality was calculated for each breeding chamber by subtracting the number of fourth-instar molt casings and number of live larvae from the number of live larvae on the previous day. Monitoring ended once all adults emerged.

### Statistical analyses of experimental data

We used Gaussian Generalized Linear Models (GLMs) to predict survivorship over time across the density gradient. We did not account for random effects due to high synchrony of development and mortality across replicates within each treatment. Developmental synchrony also demonstrated that the assumption of independent right-censorship within Kaplan-Meier plots was invalid [42]. All other life history traits of immature individuals (pupation rate, aggregate survivorship, fecundity) were reported by calculating their mean and variance. Pupation rate and overall survivorship from larvae to pupae and pupae to adult were compared with analysis of variance (ANOVA). Where significant effects occurred, comparisons were made and alpha levels were adjusted using Tukey Honestly Significant Difference (HSD) tests.

### Mathematical models

**Fig. 1:**
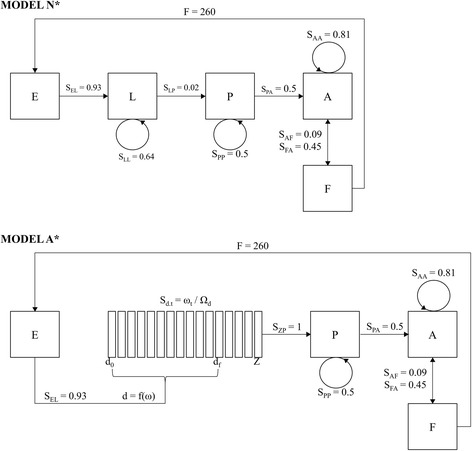
Diagrams outlining the structure of the two matrix projection models used in this study. Null Model N* (above) is an extension of a Lefkovitch day-adjusted stage structured model. Alternative Model A* (below) combines a Leslie daily change matrix model for the larval stage with a Lefkovitch day-adjusted matrix model for the other components of the mosquito life-cycle

Two models linking larval development and environmental conditions were parameterized using values from our experiments and published literature. Model N* was a stage-based matrix projection following the work of Lefkovitch et al. [43], and was developed with the objective of providing a null model responsive to extrinsic environmental factors but ignorant of the intrinsic biological factors of *Cx. quinquefasciatus* (Fig. 1). Female fecundity and fertility values were based on our field observations of egg rafts. We used average time to pupation and adult emergence across experimental treatments as estimates of daily stage change rates in the matrix. Life expectancy in adults was set to 22 days with a gonotrophic cycle of 7 days [19, 44].

Model N* predictions were contrasted with an alternative model designed to assess the competition hypothesis of the study. This alternative matrix model (Model A*) relied on density-dependent larval interactions determined by the equation:

1 $$ {\mathrm{S}}_{\mathrm{j}.\mathrm{t}}={\upomega}_{\mathrm{t}}/{\Omega}_{\mathrm{j}} $$

where the survivorship (S) of cohort j at time t is equal to the nutrients (ω) available at time t divided by a coefficient of competition (Ω) upon cohort j.

In a coeval population, Ω is directly proportional to the number of immature individuals within the environment. Eq. 1 provided the framework to translate GLM coefficients of larval mortality (μ) into consumption rate functions for Model A* by estimating larval survivorship over time within resource treatment group ω (*S*_ω_(t), Eq. 2). We fitted mortality rate models rather than observed survivorship in order to eliminate the false inclusion of stage changes as density-dependent deaths.

2 $$ {S}_{\upomega}\left(\mathrm{t}\right)=\left(1-{\mathrm{GLM}}_{\mathrm{g}}\left({\upmu}_{\upomega}\sim \mathrm{t}\right)\right)\ast 40 $$

The known value of ω informed the final consumption rate function over time, which was assumed to be exponential. The central assumption of this calculation is that density-dependent effects are occurring as a result of zero time-lag absolute resource exploitation. Individuals consume their mass-dependent daily nutrient requirement and, if this requirement cannot be met for all individuals, mortality will occur at the daily timescale to fit μ to ω. This allows for calculation of the consumption rate function C_ω_(t) for a given treatment, as follows:

3 $$ {\mathrm{C}}_{\upomega}\left(\mathrm{t}\right)=\upomega \left(\mathrm{t}\right)/{S}_{\upomega}\left(\mathrm{t}\right) $$

In a mixed age population, consumption rates vary along the predicted curve Cω(t) from metabolic age 1 through Z, after which larvae pupate. Within Model A*, we expanded the larval stage of Model N* into daily metabolic ages (d_1 - Z_) that are defined by consumption instead of explicit chronological age. The low nutrient treatment consumption function C_0.375_(t) defined the consumption rates for each metabolic age. The y-intercepts of consumption functions from the other three nutrient treatments were used to determine the relationship between nutrient availability and nutrient exploitation upon hatching, producing variable metabolic ages of first-instar larvae. The maximum metabolic age (d_f_) of first-instar larvae upon hatching was set to be the age at which C_0.375_(d_f_) was equal to the intercept C_3_(0). The final larval metabolic age, Z = 38 days, was determined by time to pupation from d_f_ = 33 days.

Within the larval sub-matrix of Model A*, daily survivorship values for each metabolic age cohort were calculated as a function of resources available. In the simulated environment, the coefficient of competition, Ω, is calculated by the summed competitive effects of all cohorts (1 through Z) on cohort j in the equation:

4 $$ {\Omega}_{\mathrm{j}}=\sum \limits_{1\to \mathrm{Z}}^{\mathrm{i}}{\mathrm{C}}_{\mathrm{i}}{\mathrm{N}}_{\mathrm{i}}/{\mathrm{C}}_{\mathrm{j}} $$

where the consumption rate (C) is treated as a proportional competitive effect, allowing the competition coefficient to represent the number of cohort-equivalent individuals competing with cohort j. This mathematical consideration favors larger cohorts by reducing the relative competitive effect exerted upon them by smaller cohorts, as is the case in natural systems [30]. Metabolic age Z permits larvae to pupate and enter the Lefkovitch matrix stages of the model that maintained structure from Model N* (e.g., adults, pupae, eggs). We termed Model A* a focal stage matrix, structured as explicit metabolic ages nested within daily-adjusted stage change probabilities. The projection cycles of Models A* and N* are shown in Fig. 1. A description of all metrics and their values can be found in Table 2. Models were constructed, projected, and analyzed using R statistical software [45].

**Table 2:** List of parameters and functions used in the models

Parameter	Description
N_t_	Population at time t
ω(t)	Resources available over time within an experimental treatment
ω_t_	Resources available within an environment at time t
C_ω_(t)	Experimentally determined consumption rate function for resource treatment group ω
C_j_	Consumption rate that determines metabolic age of cohort j
Ω_j_	Coefficient of competition showing exploitative effects of all cohorts present in an environment on cohort j
d	The metabolic cohort that first-instar larvae join upon hatching
d_f_	The highest metabolic cohort that newly hatched larvae can join
Z	The final metabolic age cohort in the model with the largest consumption rate
μ_ω_(t)	Mortality curve of a given resource treatment group ω
S_ω_(t)	Survivorship over time within resource treatment group ω
S_j.t_	The survivorship of cohort j at time t
F	Fecundity of gravid females
E	Rain event day

### Model assumptions and initial conditions

Ecologically, Model A* assumes an absolute exploitative competition response within the larval population. This means that each day, larvae will consume the amount of nutrients designated by their metabolic age that are available to them. If this immediate need cannot be met, the model then assumes that larval mortality operates on a zero time-lag response if nutrients are exhausted from the environment. Biologically, Model A* assumes a low efficiency of resource processing and mass accumulation, particularly in low nutrient environments, as seen in our analysis of in-lab observations. Low efficiency is compounded by the assumption that larvae do not eat the processed waste of other larvae and do not consume the bodies of larvae which have died as a consequence of density-dependence, upregulating the rate at which nutrients leave the simulated environment. In reference to adult populations, both models remove males through the adult sub-matrix, discounting the mating process by assuming random mixing. Model A* requires both males and females in the larval stage due to their equal contribution to competition effects.

At the simulated system scale, the model assumes consistent temporal distribution of detritus, causing rain events, even those close together in time, to load the same amount of detritus into larval habitats. For simplicity and model tractability, we generated models simulating population dynamics on a single habitat type with constant size and water-holding capability. Our modeled habitats are equivalent to roadside catch basins, as they have been well characterized in their *Culex* spp. larval dynamics and response to rainfall [39], are known to maintain high abundance of *Cx. quinquefasciatus* [37, 46], are widely distributed in urban areas of the USA, and are the most commonly targeted structure in urban larval control campaigns [9, 11, 47, 48]. The model also assumes that the effects of evaporation within the system are negligible, and that oviposition site selection of *Cx. quinquefasciatus* females is strictly adherent to catch basin habitats [23, 24].

After determining the matrix values of Models N* and A*, we simulated the projection cycles of each model with two rain events of equal size in an environment of 52 simulated catch basins that held a normal distribution with a mean 225 ± 11 g of detritus. Four kilograms of detritus were introduced to each simulated catch basin for a total of 208 kg introduced into the system. We used this value for an assumed 10 m^2^ drainage area per catch basin and consistent average of 400 g detritus/m^2^. The decay rate was set to 0.1% of matter being converted into directly consumable resources for larvae each day through bacterial decomposition [49]. The catch basin count is approximately the size of past study sites in the Atlanta area [7]. Rainfall size was assumed to be consistent for all simulations to maintain realism of simulated flushing and nutrient delivery by water collected over the large drainage area of these basins.

Simulations of single rain event systems with 200 adults were observed for timing to first peak of adults and equilibrium age distribution. In double-event simulations, an initial population distribution of 363 eggs, 592 larvae, 20 pupae and 23 adults per catch basin was selected for model runs based on typical adult resting behavior of *Cx. quinquefasciatus* individuals observed in the field (Vazquez-Prokopec et al., unpublished) and the equilibrium stage distribution from Model N*. In both models, rain events flushed eggs and pupae from the population with no time delay. We assumed two independent scenarios of the impact of rainfall on immature stages where (i) all eggs and pupae were flushed from the system or (ii) 50% of eggs and pupae were flushed. Larvae remained in the system following previous work performed on flush evasion behavior of culicine larvae [36].

Proliferation rates of Models N* and A* were quantified by mean number of adults produced per catch basin after 75 days. Paired events E_1_ = 2 and E_2_ = 2 + t were delayed on a lag ranging from t = 1 to t = 35. The mean proliferation of adults was determined for each lag and each subset of lags by week. Means were taken as a fraction of productivity from single rain event simulations E = 2. A 75 day monitoring period was selected in order to capture the first peak and decline of adult proliferation seen within the Model A* single-event simulation.

Finally, we measured sensitivity of Models N* and A* to the reduction of larvae or adults, the two targets of vector control. The same 52 catch basin system used to determine relative productivity in response to rain event delays was simulated with E_1_ = 2 and E_2_ ranging from 18 to 21. Both models included two scenarios simulating a reduction of 50 or 75% of the initial population of larvae or adults. Sensitivity was calculated by dividing the total number of adult mosquitoes that emerged in scenarios simulating a reduction in vector abundance by the total number of mosquitoes in scenarios in which vector abundance was not modified. In these sensitivity analyses, we assumed 100% flushing of eggs and pupae from the simulated environment based on literature, our results, and observations from the field [13, 36].

## Results

### Life table parameter estimation

*Culex quinquefasciatus* fecundity was, on average (± 95% CI), 258 ± 23.0 eggs. Hatch rate was, on average, 91.0 ± 6.5% and highly synchronized, occurring 2.16 ± 0.13 days after oviposition. When applied to Models N* and A*, we assumed hatch rate to be 2 days for simplicity of calculation. Life history trait parameters (survivorship, rate of stage change) of larval competition experiments are listed in Table 3. Pupal survivorship to adulthood was consistent across treatments, ranging from 93.0 to 99.0% (*F*_(3, 527)_ = 0.5; *P* = 0.73). Survivorship from the larval stage to pupation decreased along the nutrient gradient from 99 ± 1.0% in the highest nutrient environment to 23.5 ± 11.6% in the low nutrient treatment (Table 3). Strength of competitive effects was inversely related to nutrient availability, with significantly decreased larval survivorship in the two lower nutrient treatments compared to the two higher nutrient treatments (*F*_(3, 796)_ = 102.6; *P* < 0.001). The difference between mean time to pupation and mean time to emergence for all individuals within the experiments was 2.8 ± 0.4 days and did not significantly differ.

**Table 3:** Survivorship and stage change rates quantified from larval competition experiments

Nutrient treatment	Survivorship (mean ± 95% CI)^a^	Stage change rate (mean days ±95% CI)^a^
High (t_0_ = 3 mg/larva)		
Larval pupation	99.0 ± 1.0^A^	12.2 ± 3.43
Pupal emergence	98.5 ± 1.0^A^	–
Total emergence	97.5 ± 1.6	15.2 ± 3.3^A^
Mid-High (t_0_ = 1.5 mg/larva)		
Larval pupation	94.5 ± 3.9^A^	15.4 ± 7.3
Pupal emergence	94.5 ± 2.0^A^	–
Total emergence	95.0 ± 4.3	18.42 ± 7.4^AB^
Mid-Low (t_0_ = 0.75 mg/larva)		
Larval pupation	48.5 ± 7.1^B^	23.8 ± 13.2
Pupal emergence	99.0 ± 1.0^A^	–
Total emergence	51.5 ± 7.4	26.6 ± 12.6^BC^
Low (t_0_ = 0.375 mg/larva)		
Larval pupation	23.5 ± 11.6^C^	31.7 ± 14.0
Pupal emergence	93.0 ± 5.0^A^	–
Total emergence	24.0 ± 5.7	33.9 ± 12.3^C^

^a^Letter groups A-C denote significant differences between experimental treatments by HSD α < 0.05

**Fig. 2:**
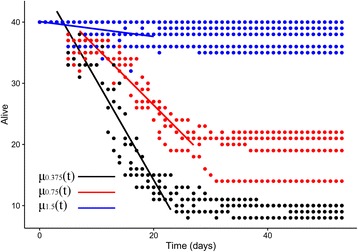
Survivorship data (points) quantified from larval competition experiments performed used to predict mortality rates (through Generalized Linear Model lines) over the period in which density-dependent effects operated. Nutrient treatments are represented by color, excluding the highest nutrient treatment in which no density dependent effects were observed

GLM-predicted mortality curves for each nutrient treatment (Table 4) showed significant associations between time and larval death in the Low through Mid-High treatments (Fig. 2). GLMs were calculated as a linear relationship between cohort mortality and time:

**Table 4:** Generalized Linear Models fitted to mortality data curves in low nutrient environments

Nutrient treatment	Slope ± SE	Intercept ± SE	Experimental day range
High (t_0_ = 3.0 mg/larva)	na	0.2 ± 0.32	na
Mid-High (t_0_ = 1.50 mg/larva)	0.3 ± 0.06*	-0.2 ± 0.75	0–20
Mid-Low (t_0_ = 0.750 mg/larva)	2.3 ± 0.09*	-12.7 ± 1.61*	7–27
Low (t_0_ = 0.375 mg/larva)	4.1 ± 0.15*	-16.6 ± 2.12*	3–23

*P < 0.001 by Gaussian GLM

*Abbreviations*: na, not applicable; SE, standard error

5 $$ {\mu}_{\omega }(t)=\mathrm{N}\ast t+\mathrm{intercept} $$

The steepest GLM slope occurred within the low nutrient treatment, as assumed by the survivorship data in Table 3, which was predicted to lose an average of 4.1 ± 0.15% of larvae each day in the period that the treatment experienced density dependent effects (days 3–23) (Table 4, Fig. 2).

**Fig. 3:**
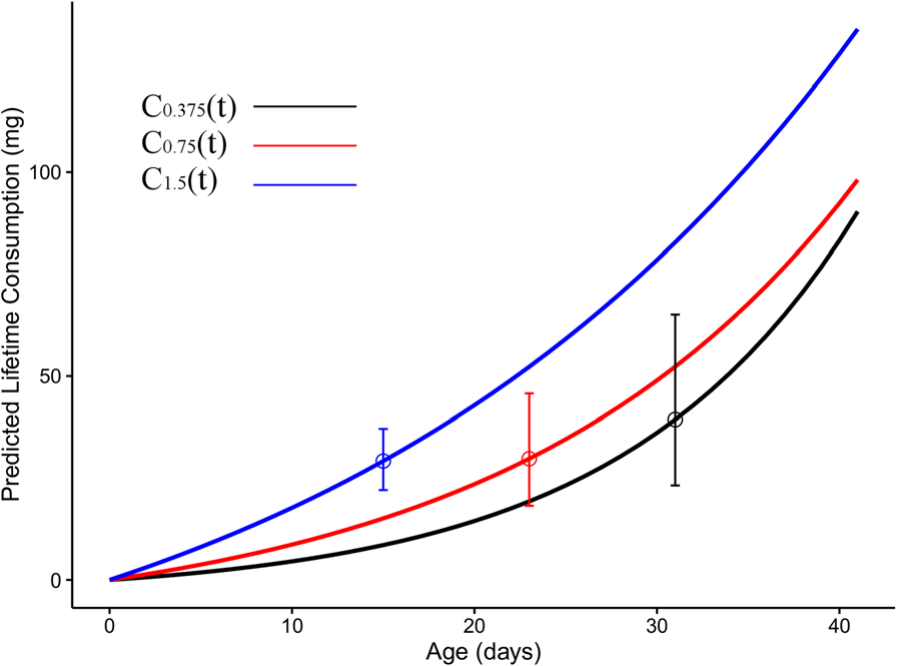
Generalized Linear Models estimated cumulative nutrient consumption trends of *Cx. quinquefasciatus* larvae across treatments (Eq. 3). Points with vertical error indicate the average day at which pupation occurred for each treatment and the range of predicted lifetime consumption for larvae in each treatment

The basal consumption rate of first-instar larvae upon hatching (i.e. the intercept of the consumption function) increased with starting per capita nutrients from C_0.375_(0) = 0.31 mg to C_1.5_(0) = 1.51 mg of daily consumption per first-instar larva. When the per capita consumption rate functions were summed over the duration of the experimental trial (Fig. 3), pupation was associated with a consistent predicted lifetime cumulative consumption of 32.8 ± 11.6 mg per larva.

### Model application

**Fig. 4:**
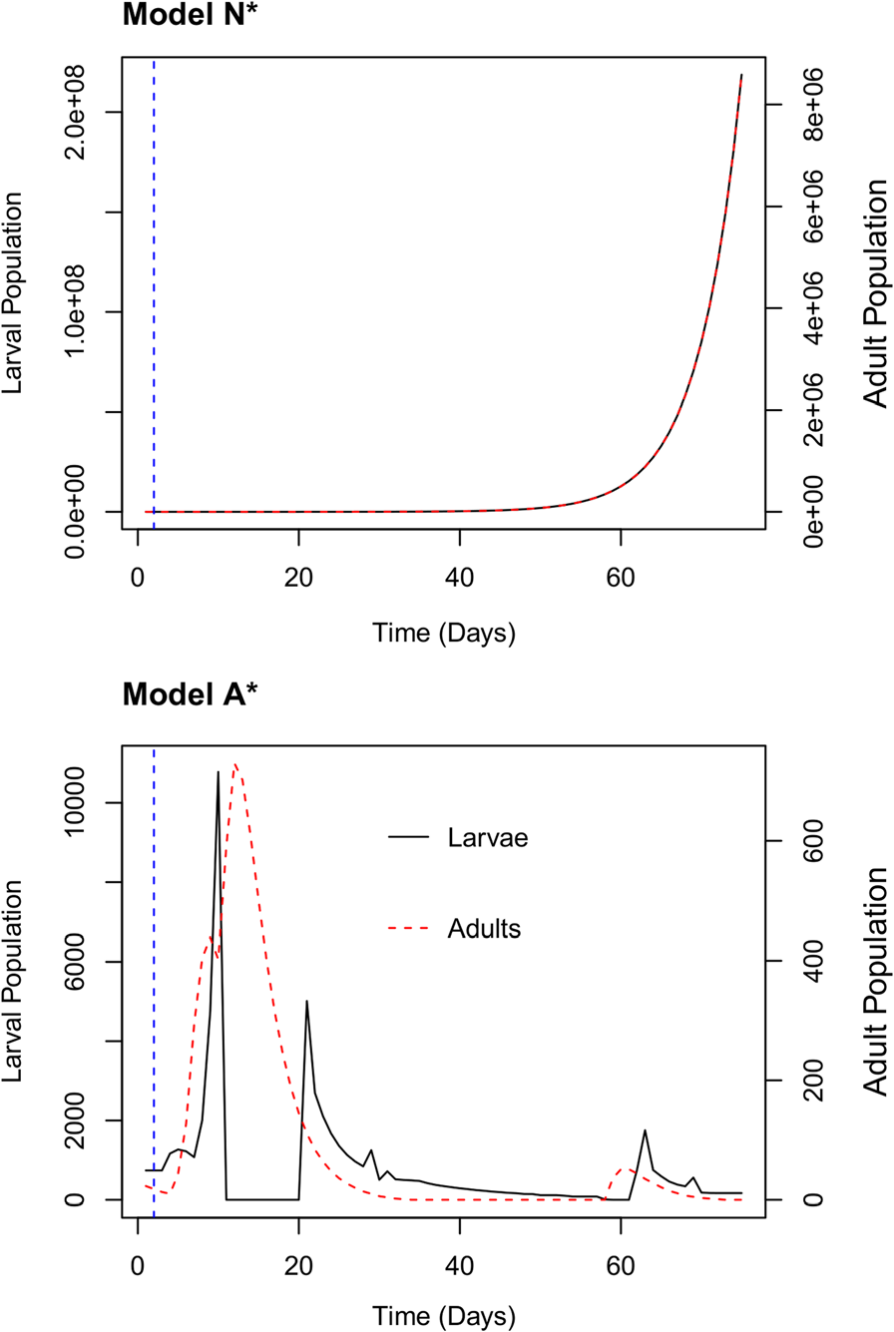
Sample runs of Models N* (above) and A* (below) with single rain event simulations at time E = 2. Model N* produces an exponential growth curve identical in adults and larvae while Model A* yields more realistic boom-bust dynamics through lagged larval and adult cycles

In Model N*, single rain events at the start of new simulated seasons with initial populations of 200 adults approached equilibrium relative distributions of 0.36 eggs, 0.59 larvae, 0.02 pupae and 0.02 adults. Upon adding intrinsic competition through Model A*, total productivity approached a parabolic shape consistent with our observed data and growth trends in urban environments (Fig. 4) [33, 34]. After resources diminished through direct decomposition and consumption by larvae, peaks of adult proliferation continued to occur with dampened larval oscillations.

**Fig. 5:**
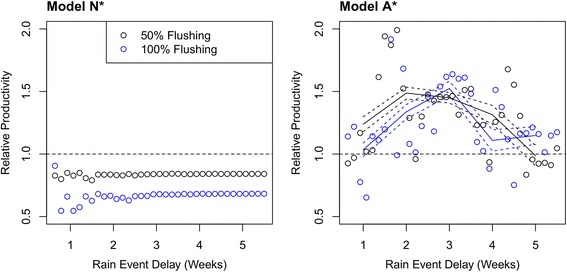
Adult productivity predicted by Model N* (left) and Model A* (right) across a gradient of rainfall periodicity (x-axis indicates the time gap in between two rain events). Relative productivity is a comparison between double-event simulations and a single-event simulation on the same first day (E = 2). Points show mean population response across 52 simulated catch basins

The equilibrium distribution of Model N* was used to define initial populations of double-event simulations for both Model N* and A*. Total productivity estimated by Model N* in double-event simulations increased exponentially towards baseline proliferation of single-event simulations only after rainfall periodicity was greater than 40 days. Variable impact of rainfall on flushing of immatures (50 or 100% mortality in eggs and pupae) produced the same qualitative trend (Fig. 5). The intensity of suppressed proliferation due to a second rainfall event was proportional to the fraction of immatures flushed out of the system. In Model A*, 3 week spacing between rain events led to consistent high proliferation distributions with 1.52 ± 0.06 increases in productivity from a second rain event (Fig. 5). When only 50% of eggs and pupae were flushed from the system, peaks in proliferation shifted to favor smaller rain delays, between 2 to 3 weeks, but were qualitatively consistent with 100% flushing scenarios. The percent change in proliferation was also quantitatively consistent, showing 1.49 ± 0.04 fold increases in productivity. Though both scenarios reflect the natural response of *Cx. quinquefasciatus* populations to rainfall periodicity, 100% flushing of eggs and pupae more closely resembles the timing of proliferation blooms observed in the field.

**Fig. 6:**
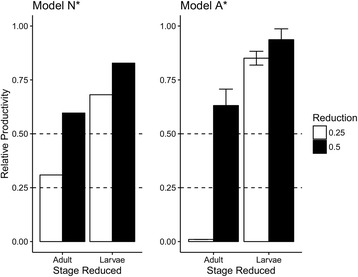
Sensitivity of Model N* (left) and A* (right) to changes in initial larval populations or adult populations. Initial populations of the specified stage were reduced to the levels shown in the legend (0.25 and 0.5) and taken as a fraction of productivity without reduction at day 75. Error bars in Model A* represent 95% CI of mean relative productivity

Sensitivity of Model N* and A* to reductions in either initial larval or adult populations showed that both were more sensitive to adult perturbation (Fig. 6). Adult reduction by 75% of the initial population yielded 69.1% reduction and halted productivity in Model N* and A*, respectively. However, larval reduction by 75% only reduced proliferation by 30.9% (N*) and 14.9 ± 3.2% (A*) of final productivity.

## Discussion

By linking field observations, experiments, and mathematical models, we provide evidence of the strong influence that environmental stochasticity and intraspecific competition have in *Cx. quinquefasciatus* populations. Rain events are a bottom-up force influencing nutrient loading and per-capita larval consumption rates. Additionally, intraspecific competition in the larval stage stabilizes population size through differential larval mortality. Together, both forces influence rates of adult productivity and explain boom-boost dynamics in *Cx. quinquefasciatus* abundance occurring after a rain event. Our study also introduces a novel deterministic population matrix model that more adequately tracks metabolic age and per-capita consumption rates, leading to more realistic *Cx. quinquefasciatus* population dynamics.

Biologically, our empirical calculations of nutrient acquisition and larval competition in *Cx. quinquefasciatus* are in agreement with previous studies [15, 18, 23, 29, 50]. Survivorship to pupation varied across treatments, with estimates of lifetime resource acquisition remaining consistent in low nutrient environments while time to pupation increased by a factor of three (Table 3). Nutrient loss due to metabolism in a prolonged larval stage would lead to reduced vector body size and ultimately reduced fecundity upon emergence [19]. Thus, we propose that a multiplicative effect of decreased survivorship and decreased fecundity in low nutrient environments have the potential to limit population growth in *Cx. quinquefasciatus*. When incorporated into our mathematical model, such interactive effects were key in explaining simulated boom and bust dynamics and the observed population response of *Cx. quinquefasciatus* to rain events.

During the stable population period of single-event simulations (i.e. after the initial larval bloom), per capita survival to pupation was higher than in the volatile, exponential growth period of colonization, suggesting increased survivorship and metabolic efficiency at lower abundance or at high nutrient levels. Knowledge of population age distribution within a system and how that system responds to nutrient loading is therefore critical to understanding population response to environmental disturbance regimes or larvicide application. Insecticide-based larvicides (e.g. those that directly lead to larval mortality, such as *Bacillus thuringiensis*, bti) that present low efficacy on larval mortality in a catch basin [9, 11, 48] could be relieving the population from strong competitive pressure, leading to an initial reduction in productivity but a rapid bounce-back of populations after rain events flush or dilute chemicals in catch basins. Conversely, insect growth regulators (e.g. pyriproxyfen), which reduce adult emergence and not larval survival, would impact two components of larval dynamics: limiting adult emergence and maintaining high density-dependent mortality in the larval stage [51]. Such a relationship is supported through our investigation of Model A* sensitivity to adult and larval perturbation. The interaction between competition release and insecticide regime efficacy, though little studied, can have high value in informing vector control strategies relying on larval control [1, 9, 11, 48, 52].

Multiple vector control approaches are implemented to control WNv vectors in the USA [8, 9, 11, 48, 53–58]. While adulticiding is generally used in response to widespread human transmission or elevated mosquito infection rates, larviciding can be implemented both preventively and in combination with adulticiding. Larvicidal efficacy suffers from low residual power, leading to a need for frequent reapplication [9, 11, 47]. Our study suggests that formulations that do not alter population density but influence adult emergence will have a higher impact in culicines. Furthermore, designing novel molecule delivery modes that can endure rainfall flushing and rapidly dissolve in the water column will likely impact the population increase observed following rain events. Given the challenges that adulticiding approaches face in urban areas of the USA [54–56], effective vector control will depend on integrated management plans that utilize well-implemented larviciding as an important tool for reducing vector abundance and preventing WNv spillover. Alternatively, devising approaches to modify roadside catch basin structures to make them mosquito-free, such as those described in Souza et al. [59], can be another approach to impact rapid *Cx. quinquefasciatus* adult proliferation in urban areas.

We acknowledge several limitations of our study. Although the inclusion of density-dependent mechanisms (Model A*) recreated patterns of environmental response described in the literature, we are aware of other potential factors that can influence such population behavior. In addition to the influence of detrital loading on larval populations, natural systems are sensitive to temperature effects and rainfall intensity. Unaccounted for in the simulated system, these parameters may influence the speed and response of populations to environmental stochasticity. Future work will aim to expand our modeling effort to include more realistic environmental drivers (temperature-driven development and consumption rates) as well as a tractable epidemiological link to our purely entomological models.

## Conclusions

Driven by detrital loading through stochastic rain events, density-dependent interactions may be overlooked due to the high larval population levels found within *Cx. quinquefasciatus* larval habitats. Our data and models demonstrate that such density-dependent effects can have dramatic population-level impacts, driving larval productivity and adult *Cx. quinquefasciatus* population response to rain events. Our study therefore provides a theoretical platform for the study of immature populations and opens the door for more explicit explorations of the impact of larval and adult control in this medically relevant disease vector.

## Additional file

Additional file 1: Annotated code performed in R and used to generate all models presented in this article. (R 30 kb)

## Abbreviations

ANOVA: Analysis of variance test
GLM: Generalized linear model
HSD: Tukey’s honestly significant difference test
SE: Standard error
SLEv: Saint Louis encephalitis virus
WNv: West Nile virus
